# Mechanisms of endosperm initiation

**DOI:** 10.1007/s00497-016-0290-x

**Published:** 2016-07-23

**Authors:** Philip Hands, David S. Rabiger, Anna Koltunow

**Affiliations:** CSIRO Agriculture and Food, Commonwealth Scientific and Industrial Research Organization, Private Bag 2, Glen Osmond, SA 5064 Australia

**Keywords:** Female gametophyte, Fertilization, Endosperm, Development

## Abstract

*****Key message***:**

**Overview of developmental events and signalling during central cell maturation and early endosperm development with a focus on mechanisms of sexual and autonomous endosperm initiation.**

**Abstract:**

Endosperm is important for seed viability and global food supply. The mechanisms regulating the developmental transition between Female Gametophyte (FG) maturation and early endosperm development in angiosperms are difficult to study as they occur buried deep within the ovule. Knowledge of the molecular events underlying this developmental window of events has significantly increased with the combined use of mutants, cell specific markers, and plant hormone sensing reporters. Here, we review recent discoveries concerning the developmental events and signalling of FG maturation, fertilization, and endosperm development. We focus on the regulation of the initiation of endosperm development with and without fertilization in *Arabidopsis* and the apomict *Hieracium*, comparing this to what is known in monocots where distinct differences in developmental patterning may underlie alternative mechanisms of suppression and initiation. The Polycomb Repressive Complex 2 (PRC2), plant hormones, and transcription factors are iteratively involved in early fertilization-induced endosperm formation in *Arabidopsis*. Auxin increases and PRC2 complex inactivation can also induce fertilization-independent endosperm proliferation in *Arabidopsis*. Function of the PRC2 complex member *FERTILIZATION-INDEPENDENT ENDOSPERM* and two loci *AutE* and *LOP* are required for autonomous endosperm development in apomictic *Hieracium*. A comparative understanding of cues required for early endosperm development will facilitate genetic engineering approaches for the development of resilient seed crops, especially if an option for fertilization-independent endosperm formation was possible to combat stress-induced crop failure.

## Introduction

Seed endosperm provides a globally critical food supply via either direct human consumption or indirectly as animal feed. While great morphological diversity can be seen amongst mature seeds, the patterns and processes of Female Gametophyte (FG) and early seed development show high levels of conservation across the angiosperms. The developing seed is comprised of three distinct compartments, the embryo, endosperm, and surrounding seed coat. Embryogenesis is dependent upon accompanying endosperm development, and both compartments require an individual fertilization to initiate development but some species, termed apomicts, have evolved mechanisms to avoid the requirement of double fertilization. These species are capable of seed development with only a single fertilization event, specifically in the endosperm (pseudogamous apomicts), or in the complete absence of fertilization (autonomous apomicts).

Seed development begins with development of the ovule, a specialized structure within the ovary housing the FG. The Polygonum-type FG, evident in both eudicots and monocots and most prevalent amongst the angiosperms, is the focus of this review. This FG type is a seven-celled eight-nucleate structure comprised of four cell types including the egg and Central Cell (CC) which upon fertilization develop into the embryo and endosperm, respectively. Surrounding the FG are the ovule integuments which develop into the seed coat upon fertilization. The FG also contains the two synergid cells and three antipodal cells. The synergids play multiple roles in the fertilization process, primarily in the attraction of the pollen tube, which transmits the male gametes to the FG. Antipodal cell functions are poorly understood although in some species such as maize they are suggested to play a role in supplying nutrients to the gametophyte or developing seed (Chettoor and Evans 2015).

**Fig. 1:**
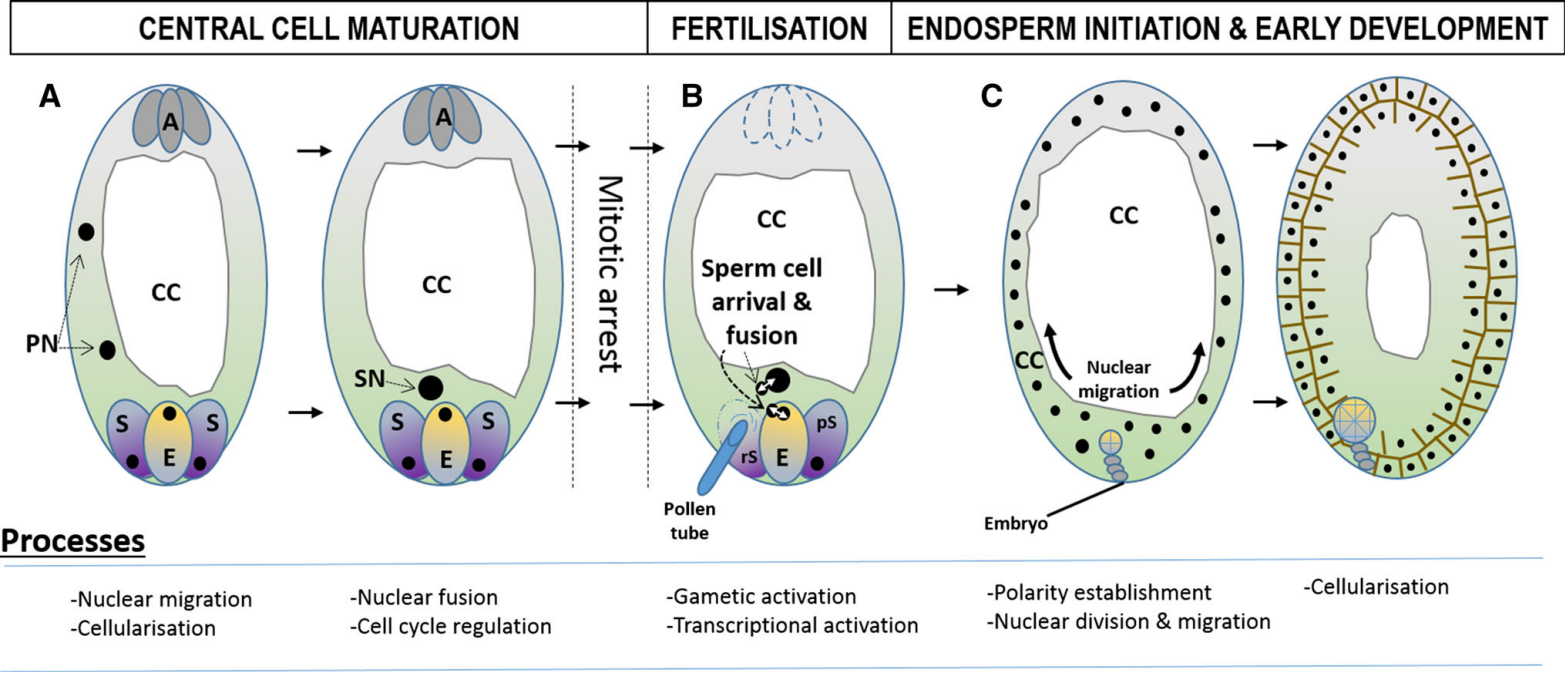
*Arabidopsis* female gametophyte and early endosperm development. Processes and events of *Arabidopsis* FG maturation, endosperm initiation, and early development. *A* Following FG cellularization, the two PN are sequestered to the CC and migrate towards the egg cell. Upon meeting the PN fuse to generate the SN positioned adjacent to the egg cell nuclei. PN fusion is the final event of FG development after which the SN is mitotically arrested until fertilisation. *B* Fertilization. Pollen tube penetration of the receptive synergid triggers cell death. Rupture releases the sperm cells which individually fuse with egg and CC. In the CC, nuclear proliferation begins rapidly after fertilization. Antipodal cell degeneration begins at this stage along with cytoplasmic fusion of the persistent synergid with CC. *C* Endosperm development. Rapid acytokinetic mitosis in the CC occurs accompanied by nuclear migration from the mycropylar to chalazal pole. CC and vacuole enlarge rapidly resulting in nuclei distributed around the periphery of the CC. Cellularization occurs with ongoing mitosis progressing from the CC periphery towards the centre until complete. Key process occurring during each stage are listed in diagrams below. *PN* polar nuclei, *SN* secondary nucleus, *S* synergid cell, *rS* receptive synergid, *pS* persistent synergid, *E* egg cell, *A* antipodal cells, *CC* central cell, *FG* female gametophyte

Polygonum-type FG development begins with meiosis of the megaspore mother cell to produce four haploid megaspores, three of which die. The surviving chalazal-most functional megaspore undergoes megagametogenesis that involves a period of syncytial development followed by cellularization to produce the typical seven-celled structure depicted in Fig. 1a. Cellular identity and organization of the developing FG is determined by the establishment of distinct polarities and nuclear positioning at the time of cellularization (Sprunck and Gross-Hardt 2011). Two of the eight nuclei (Polar Nuclei; PN) are sequestered within the highly metabolically active CC where they either partially or fully fuse prior to fertilization to form a homodiploid Secondary Nucleus (SN).

Double fertilization is initiated by delivery of the sperm cells by the pollen tube (Fig. 1b). One sperm cell fuses with the egg cell, while the second fuses with the CC, initiating embryo and endosperm development, respectively. As a consequence, the endosperm typically has a 2:1 maternal/paternal genome ratio in contrast to the 1:1 ratio of the embryo. In most flowering plant species, early endosperm development also proceeds syncytially, much like the preceding FG development, with the number of nuclear divisions typically correlating with seed size (Fig. 1c; Olsen 2004). The syncytial endosperm cellularizes and enters a maturation process accumulating storage molecules including proteins, lipids, and starches to support embryo development and growth. Dependent upon species, the endosperm remains as a persistent structure at maturity comprising the bulk of the seed, as observed in monocots such as cereals, or is ephemeral, being consumed by the enlarging embryo as seen in eudicots such as *Arabidopsis* and legumes.

Asexual seed production, termed apomixis, has been recorded in more than 400 genera across 40 separate families (Ozias-Akins and van Dijk 2007). Apomixis is comprised of three distinct developmental components; apomeiosis, parthenogenesis, and mechanisms promoting successful endosperm formation. Apomeiosis is the generation of a cell capable of forming an embryo without prior meiosis which may be either sporophytic, arising from differentiating nucellus or integument cells or, more commonly, gametophytic, originating from an embryo sac cell that has not undergone meiosis. Parthenogenesis, or spontaneous embryo development from this cell, follows and is necessarily accompanied by endosperm production to support seed development which may be either sexually or autonomously initiated. Seeds formed via apomixis germinate seedlings that are clones of the maternal parent and offer an attractive method of preserving superior genotypes in agriculture. Strategies to harness apomixis in breeding programs for the preservation of complex traits including hybrid vigour via seed and to engineer resilient seed crops and ensure endosperm formation have been discussed in recent reviews (Kandemir and Saygili 2015; Barcaccia and Albertini 2013; Koltunow et al. 2013).

Embryo formation without fertilization is common to all apomictic species, but CC fertilization is still required in most for endosperm development, a process termed pseudogamy. The capacity for autonomous endosperm development is rare (Bicknell and Koltunow 2004). In the apomictic species *Hieracium* and *Taraxacum,* seed development is initiated without fertilization and proceeds in the complete absence of paternal genetic contribution. The capacity to complete endosperm development and support seed germination independently is a key trait to understanding and engineering true apomixis in crop species. *Hieracium* is a developed model for the genetic and molecular analysis of apomixis and is well equipped for the analysis of autonomous endosperm formation with the availability of mutants and informative recombinants where the trait has segregated from apomeiosis and parthenogenesis (reviewed in Bicknell and Koltunow 2004; Koltunow and Grossniklaus 2003; Koltunow et al. 2013; Ogawa et al. 2013).

Although the cytological events surrounding double fertilization and early endosperm development were discovered more than a century ago (Nawaschin 1898; Guignard 1899) the molecular mechanisms regulating this process are only recently beginning to be understood. In this review, we focus upon the developmental window spanning CC maturation and early endosperm development with particular focus on recently discovered mechanisms regulating fertilization and endosperm initiation. We also discuss their relationship to the mechanisms of endosperm initiation and development in *Hieracium*, an autonomous apomict.

## Central cell maturation and mitotic repression

The CC comprises the bulk of the FG with a large central vacuole and cytoplasmic volume at maturity. During maturation, the PN migrate towards the CC micropylar pole into a position adjacent to the egg cell nucleus (Fig. 1a). In *Arabidopsis* and other dicots, the PN fuse to form a morphologically distinct homodiploid Secondary Nucleus (SN), whereas in most monocots, such as maize, the PN only partially fuse and remain so until arrival of the sperm cell (Willemse and van Went 1984; Huang and Russell 1992). Adjacent positioning of the egg and CC nuclei enables efficient sperm cell delivery during fertilization, and improper nuclear location may have impacts upon fertilization and seed viability (Sprunck and Gross-Hardt 2011). Little is known about the mechanisms guiding PN or SN movement and maintenance of their position prior to fertilization.

As it reaches maturity, the CC becomes mitotically arrested prior to fertilization. Cell-cycle synchrony between male and female gametic cells is required at the time of fusion for the initiation of both embryo and endosperm development (Tian et al. 2005; Russell and Jones 2015). Plant sperm cell nuclei are thought to be progressing across G1 and G2 phases at the time of fusion, whereas the female gametes are mitotically arrested prior to fertilization (Friedman 1999; Krishnamurthy 2015). In *Arabidopsis*, observations suggest that the female gametes arrest at differing cell-cycle stages with the CC at G2 and the egg cell at G1 (Berger et al. 2008; Russell and Jones 2015). The synchrony of cell-cycle phase between the sperm and CC nuclei at fertilization may explain the observed immediate onset of mitotic endosperm development, whereas mitotic divisions within the zygote are delayed (Berger et al. 2008). However, the phase of cell-cycle arrest in most angiosperm female gametes has yet to be conclusively determined.

Although mitotically arrested, the CC exhibits high levels of metabolic activity, extensive endoplasmic reticulum and is rich in mitochondria, starch and lipid reserves (Liu et al. 2010b). These observations suggest that even prior to the initiation of endosperm development, the CC is primed for its immediate initiation upon sperm cell delivery. This is supported by observations in mutations affecting the *Arabidopsis* fertilization-independent seed (FIS) polycomb repressive complex 2 (PRC2) where CC proliferation initiates in the absence of fertilization. The *Arabidopsis* FIS-PRC2 complex comprises the C2H2 zinc-finger FERTILIZATION-INDEPENDENT SEED 2 (FIS2), the SET-domain protein MEDEA (MEA; Grossniklaus et al. 1998; Kiyosue et al. 1999; Luo et al. 1999), the WD-40 protein FERTILIZATION-INDEPENDENT ENDOSPERM (FIE; Ohad et al. 1996), and the p55-like MULTICOPYSUPPRESSOR OF IRA1 (MSI1; Kohler et al. 2003; Guitton et al. 2004), and functions in the CC to deposit H3K27 repressive marks on chromatin to repress gene transcription and CC proliferation in the absence of fertilization (Köhler and Makarevich 2006; Rodrigues et al. 2010a; Xiao and Wagner 2015). Although mutations in FIS-PRC2 complex genes result in fertilization-independent endosperm initiation, the endosperm fails to cellularize, and viable seeds are not produced, likely due to its requirement in other downstream processes including endosperm cellularization and patterning (Schmidt et al. 2013; Ingouff et al. 2005; Rodrigues et al. 2010a; Hehenberger et al. 2012). Whether the PRC2 is required to maintain CC fate in other species is less clear. Downregulation of FIE orthologues in *Hieracium* and maize does not induce fertilization-independent endosperm development (Rodrigues et al. 2008, 2010b), whereas some proliferation was observed in rice (Li et al. 2014). One possibility to explain these differences is that although the PRC2 complex is conserved in a range of species, its temporal and spatial functions may have evolved differently among plants. For example, orthologues of *FIS2* and *MEA* have not been identified outside of Brassicaceae, and the composition and function of PRC2 complex members within the CC are little studied outside of *Arabidopsis* (Rodrigues et al. 2010a). However, it is also possible that differences observed in *FIE* orthologue downregulation experiments amongst species may arise from incomplete suppression via RNAi and may not reflect true loss of function phenotypes.

## Fertilization

**Fig. 2:**
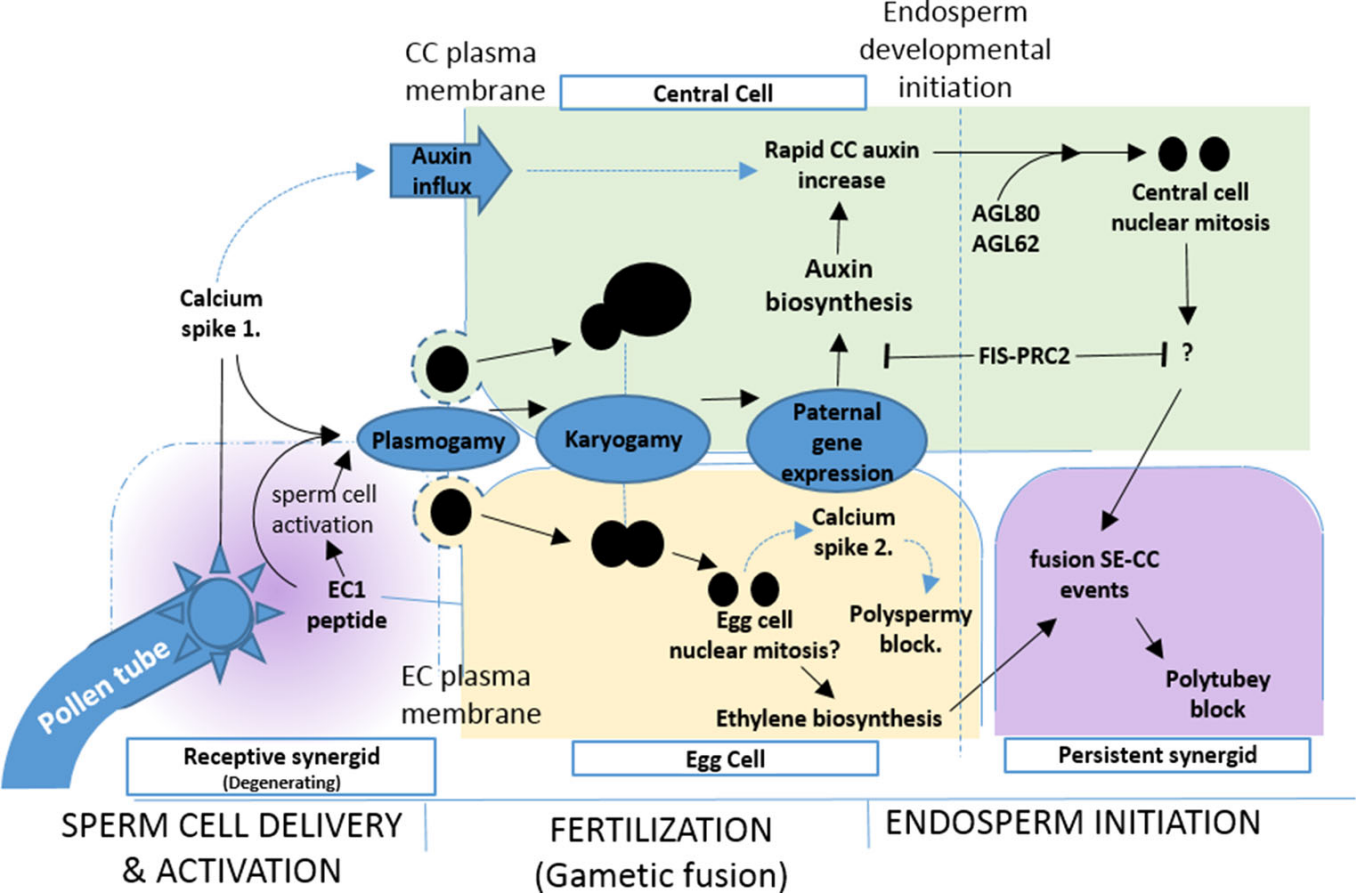
Diagram of signalling events of *Arabidopsis* fertilisation and endosperm initiation. Procession of the events and signalling involved in fertilization and endosperm developmental initiation in *Arabidopsis*. Pollen tube rupture in the degenerating synergid releases the sperm cells. Associated calcium signalling triggers the release of EC1 peptides involved in sperm cell activation and potentially auxin influx to the CC. Individual sperm cells fuse with E and CC (Plasmogamy) followed by fusion of their respective nuclei (Karyogamy). In the CC maternal auxin biosynthesis, genes are repressed by the FIS-PRC2. Nuclear fusion in the CC initiates paternal gene expression, which results in auxin biosynthesis and increase in the CC that may also include auxin influx. In conjunction with AGL62 activity, increased auxin concentration triggers the release of mitotic arrest mechanisms and nuclear proliferation in the CC. Nuclear fusion in the egg cell triggers calcium signalling associated with rapid polyspermy blockade along with ethylene biosynthesis responsible for triggering plasmogamy of the persistent synergid with the CC and polytubey blockade. *Solid black arrows* indicate reported signalling events. *Blue-dotted arrows* represent proposed signalling events

Fertilization is comprised of multiple orchestrated and often recurring regulatory processes (Fig. 1b). Figure 2 depicts events during the three stages of fertilization: sperm cell delivery and activation, gametic fusion, and initiation of embryo and endosperm development, with a focus on knowledge gleaned from *Arabidopsis*. Fertilization begins with arrival of the Pollen Tube (PT) to one of the two synergid cells, termed the receptive synergid. PT guidance to the receptive synergid is controlled by maternal sporophytic tissues and intricate signalling mechanisms involving secretion of attractant peptides, such as LURE1, by the synergid cells (Okuda et al. 2009). Interactions between the receptive synergid and the PT are mediated via the RALF/FERONIA small peptide signal transduction pathway coupled to Ca^2+^ oscillations (Ngo et al. 2014) within the synergid, and induce PT burst and concomitant receptive synergid degeneration. The complex regulation of PT guidance and sperm cell release are summarized in recent reviews (Bleckmann et al. 2014; Dresselhaus and Franklin-Tong 2013; Lausser and Dresselhaus 2010; Marton and Dresselhaus 2010; Dresselhaus et al. 2016). Following release, sperm cells undergo rapid, directed movement to a position adjacent to the egg and CC nuclei. As plant sperm cells are immotile, it is unclear whether their movement occurs simply via cytoplasmic flow associated with PT rupture or is under greater maternal influence, such as via elastic deformation and differential surface interactions from the surrounding egg and CC. At this time, sperm cells also become activated for fusion with the female gametic nuclei (Sprunck et al. 2012). Subsequent fertilization is dependent upon two distinct processes, plasmogamy (cytoplasmic fusion) delivering a single sperm cell nucleus to the egg and CC followed by karyogamy (nuclear fusion) to initiate development.

Following PT rupture, two transient Ca^2+^ signalling events occur which appear to be involved in sperm cell activation and the prevention of polyspermy in *Arabidopsis*. The first of these events is associated with PT rupture and appears to be required for sperm cell activation, when a short cytosolic Ca^2+^ transient is observed in both the egg and CC (Hamamura et al. 2014; Denninger et al. 2014). Activation furnishes the gametes with a competency for nuclear fusion and is critical in reproductive development to circumvent innate mechanisms inhibiting fusion. This initial Ca^2+^ transient coincides with the exocytosis of EGG CELL 1 (EC1) peptide from the egg cell. EC1 is a cysteine-rich peptide (CRP) shown to regulate sperm cell activation via redistribution of the fusion-critical GENERATIVE CELL SPECIFIC 1 (GCS1) protein, also named HAPLESS 2 (HAP2), to the nuclear surface of the sperm cell (Sprunck et al. 2012; Mori et al. 2006; von Besser et al. 2006). The fertilized egg and CC of *gcs1/hap2* mutants show normal nuclear adhesion but no membrane fusion, indicating that this protein functions as a potential fusogen (Mori et al. 2006). A similar function for this protein in protozoa and algae suggests conservation of the mechanism amongst eukaryotes (Wong and Johnson 2010; Mori et al. 2015). Sprunck et al. (2012) have shown that sperm cell arrival in the FG triggers exocytosis of EC1-containing vesicles from the egg cell essential for successful fertilization, although details of the precise mechanism of gametic activation remain unclear and suggest additional factors are involved. Igawa et al. (2013) report that the sperm cell plasma membranes remain intact prior to plasmogamy raising questions on the pathway of the apoplastic EC1 peptide signal to the sperm cell nucleus. It is currently unclear what, if any, function the CC calcium transient has in this process or whether it has a separate function.

The second Ca^2+^ transient is observed only in the egg cell following successful fertilization and is thought to be involved in the prevention of polyspermy, consistent with observations of similar function in animal cells (Denninger et al. 2014; Scott et al. 2008). Polyspermy-blocking mechanisms provide a rapid, short-term blockade to fertilization of an individual gamete by more than one sperm cell. Although mechanisms of polyspermy blockade in plants remain unclear, maize egg cells develop thicker cell walls within 30 s of fertilization (Kranz et al. 1995) and in *Chlamydomonas* GCS1/HAP2 and FUS1, another membrane fusion-associated protein, are degraded rapidly after fusion (Liu et al. 2010a). Both of these events are potentially associated with preventing further gametic fusions, but a direct functional link between these or any other events and calcium signalling remains to be demonstrated. Notably, however, polyspermy-blocking mechanisms do not appear to be active in the *Arabidopsis* CC. Fertilization with pollen of the *Arabidopsistetraspore* mutant, a male meiotic mutant producing additional sperm cells that are released simultaneously from one pollen tube (Spielman et al. 1997; Kong et al. 2015), typically results in multiple CC fertilizations and polyploid endosperm but the embryo is not affected (Scott et al. 2008). The absence of the second Ca^2+^ spike in the CC raises questions therefore about the distinction of the CC fertilization requirements and process in comparison to the egg cell.

During fertilization in *Arabidopsis,* PT rupture induces degeneration of the receptive synergid while the persistent synergid remains functional in PT attraction and is thought to function as a backup mechanism, allowing attraction of a second PT (Polytubey) in the event of a failed fertilization (Maruyama et al. 2013). Following successful fertilization, the persistent synergid is eliminated to prevent arrival of additional pollen tubes. In *Arabidopsis*, polytubey blockade is regulated by both egg and CC independently and synergistically, with this dual control over PT attraction likely providing a fitness advantage by the recovery of failed individual fertilizations via heterofertilization (Maruyama et al. 2013). Interestingly, in *Arabidopsis*, the polytubey blockade also appears to involve FIS-PRC2 function within the CC, with mutations affecting any of the components displaying a polytubey phenotype (Maruyama et al. 2013). Maruyama et al. (2015) went on to show that elimination of the persistent synergid involves a cytoplasmic fusion event with the CC (SE fusion). SE fusion is triggered by CC fertilization, resulting in the rapid dilution of PT attractants contained within the persistent synergid; however, the synergid and CC nuclei do not undergo karyogamy. Instead, elimination of the synergid nucleus is induced via ethylene signalling pathways originating from the fertilized egg cell, in conjunction with the FIS-PRC2 activity within the CC.

The processes of nuclear fusion during fertilization share many similarities to those of the preceding PN fusion, and several mutants affecting PN fusion have been isolated (Pagnussat et al. 2005; Portereiko et al. 2006). The yeast immunoglobulin-binding protein (BiP) functions in nuclear membrane fusion during mating (Noh et al. 2003). At least three *Arabidopsis* BiP orthologues are identified and shown to function in both PN and sperm-SN nuclear fusion with important effects upon fertilization and endosperm initiation (Maruyama et al. 2010, 2015). In these mutants, fertilization often results in sperm cell fusion with unfused PN and subsequent abnormal and asynchronous endosperm nuclear proliferation, failed cellularization and seed abortion that is likely due to the subsequent diploid rather than triploid constitution (Maruyama et al. 2010). The signalling and regulation of nuclear fusion is a complex and critical component of reproductive development, the details of which are only beginning to be elucidated.

## Endosperm initiation and early development

Endosperm development initiates almost immediately after CC fertilization and in most angiosperms develops syncitially before cellularizing. Figueiredo et al. (2015) recently uncovered a role for auxin in the induction of endosperm proliferation in *Arabidopsis* showing that auxin levels are sufficient to override FIS-PRC2 suppression of SN proliferation. Auxin is well established as an important regulator of cell-cycle phase progression and expression of key cell-cycle genes (John 2007). In the *Arabidopsis* CC and endosperm, the FIS-PRC2 complex represses maternal expression of *TAR1* and *YUC10*, both of which have critical functions in auxin biosynthesis (Figueiredo et al. 2015, Zhao 2014). Following fertilization, a rapid increase in auxin signalling is observed within the early endosperm, whereas prior to fertilization auxin signalling is not detectable. The authors propose that paternal expression of auxin biosynthesis genes following fertilization promotes the initiation of endosperm development in *Arabidopsis*. Homologues of auxin biosynthetic genes are also shown to be paternally expressed in rice and maize endosperm suggesting that this signalling mechanism may be conserved amongst the angiosperms. Interestingly, polar auxin transport is found to be dependent upon Ca^2+^ availability via effects upon plasma membrane ATPase activity and apoplastic proton abundance (Vanneste and Friml 2009), suggesting that auxin influx may also be a component of the CC auxin increase following fertilization triggered by the initial calcium transient detected in both the egg and CC. Auxin has also been shown to function in the regulation of early endosperm development in maize and *Arabidopsis*, where plants deficient in auxin biosynthesis and signalling processes show impaired endosperm nuclear mitosis and migration (Bernardi et al. 2012; Forestan et al. 2010).

Nuclear mitosis and migration during the syncytial development phase and timing of the transition to cellular development play an important role in determining endosperm size and seed viability (Hehenberger et al. 2012). Rapid division and nuclear migration, in parallel with expansion of a large central vacuole, distribute the dividing nuclei around the periphery of the developing endosperm cavity prior to cellularization (Fig. 1c). This pattern of nuclear migration is described in several species including *Arabidopsis*, barley, wheat, and maize and is important to subsequent functional differentiation although few common regulators between species have been identified. Cell-cycle regulation is critical to syncytial endosperm development where signals maintaining mitotic synchrony allow rapid progression with important consequences to final seed size and weight (Dante et al. 2014; Sabelli and Larkins 2009). To date, relatively little is known about the molecular factors and signals responsible for this regulation and many of those identified are shared with endoreduplication mechanisms. The rice *ENDOSPERMLESS1* (*ENL1*) was recently identified as a syncytial endosperm specific developmental regulator encoding an SNF2 helicase protein involved in karyokinesis essential to rapid nuclear cycling during syncytial development (Hara et al. 2015). Mutants show failed endosperm development during the syncytial stage and differ significantly to an *Arabidopsis* orthologue mutant, which shows only minor effects. It is well established that the RBR1-E2F pathway, a master cell-cycle regulator, has a critical role in cell-cycle activity during endosperm development (Ebel et al. 2004; Dante et al. 2014). Analyses have identified differences in cell-cycle control mechanisms between monocots and dicots where increased complexity of the *RBR* family in monocots has potentially allowed functional diversification (Lendvai et al. 2007). The maize *RBR1* homologue regulates key aspects of endosperm development, and its function is shown to be coupled to that of *CDKA;1* in regulating endosperm endoreduplication (Sabelli et al. 2013). Recently, Sornay et al. (2015) reported that deregulation of *CYCD* gene activity affects syncytial development in *Arabidopsis*, potentially via inactivation of RBR proteins resulting in released mitotic suppression in the CC and, ultimately, partial abortion of seed development. Mizutani et al. (2010) have also shown that *KRP3*, a rice cyclin-dependent kinase inhibitor is specifically expressed during the syncytial phase of endosperm development and suggest its involvement in syncytium-specific cell-cycle control.

Small peptide signalling pathways are also thought to play multiple roles in coordinating seed and endosperm development with many signalling peptide-encoding genes found to be differentially expressed at separate developmental stages and fertilization (Huang et al. 2015; Ingram and Gutierrez-Marcos 2015). *CLE8* and *CLE19*, both members of the *CLAVATA3/EMBRYO SURROUNDING REGION* (*CLE*) family, are shown to affect *Arabidopsis* endosperm development. Plants carrying a CLE19 antagonistic construct had delayed nuclear proliferation and reduced nuclear number at the time of cellularization, along with prolonged expression of *MEA*, *FIS2* and *AGL62* (Xu et al. 2015). Interestingly, *CLE19* expression in the seed is restricted to the embryo, suggesting function as an embryo-derived signal that regulates endosperm development (Xu et al. 2015). *CLE8* is the only member of the family found to be exclusively expressed in seed tissues and positively regulates expression of *WOX8* (*WUSCHEL*-*LIKE HOMEOBOX8*), a transcription factor shown to promote seed growth and size. Downregulation of *CLE8*, which is expressed in the embryo and surrounding endosperm region, results in reduced nuclear proliferation, defective early nuclear migration and disorganization of the nuclear endosperm, and reduced seed size (Fiume and Fletcher 2012).

Timing of the transition from syncytial to cellular endosperm development is an important determinant of seed size and viability (Hehenberger et al. 2012). In *Arabidopsis*, the type-I MADS-box transcription factor AGL62 plays a prominent role in timing of the syncytial-cellular developmental transition. *AGL62* expression dosage affects the timing of cellularization with mutation of this gene causing premature endosperm cellularization, typically after only a few divisions (Kang et al. 2008). Mutations affecting members of the *HAIKU* (*IKU*) pathway also affect syncytial development via cell-cycle regulatory effects which reduce the number of nuclear divisions prior to cellularization in *Arabidopsis* (Luo et al. 2005). The IKU pathway member *MINISEED3*, a WRKY transcription factor, is shown to directly regulate the expression of the cytokinin oxidase gene *CKX2*, suggesting that the restriction of cytokinin signalling within the endosperm is important for promoting syncytial development (Li et al. 2013). Both *AGL62* and *CKX2* are reported to be direct targets of the FIS-PRC2, which itself is also required for endosperm cellularization where fertilized CCs of FIS-class mutants initiate syncytial endosperm development but fail to undergo cellularization and seeds subsequently abort (Ohad et al. 1996; Chaudhury et al. 1997; Kang et al. 2008; Hehenberger et al. 2012; Li et al. 2013). Downregulation of FIE orthologues in rice and *Hieracium* also affects cellularization (Li et al. 2014; Rodrigues et al. 2008), suggesting some conservation for the role of the PRC2 in regulating endosperm syncytial development.

## Autonomous endosperm development in *Hieracium*

While apomixis itself is relatively common amongst the flowering plants, the inclusion of autonomous endosperm development as a component of that reproductive process is rare (Koltunow and Grossniklaus 2003). *Hieracium* is one of the very few species showing natural autonomous endosperm development and are completely independent of fertilization for seed formation. At least three genetic loci are shown to be involved in apomixis in *Hieracium* species. Deletion mutagenesis has identified two loci responsible for the apomeiosis and parthenogenetic components of apomictic reproduction, loss of apomeiosis (*LOA*) and loss of parthenogenesis (*LOP*); Catanach et al. 2006; Koltunow et al. 2011). In a separate investigation, Ogawa et al. (2013) were able to show that autonomous endosperm production can be uncoupled from the other components of apomixis in *Hieracium* and appears to be regulated by a third genetic locus (*AutE*). Specific genes within these loci have not been identified.

The observation of autonomous endosperm development in *Arabidopsis* FIS-PRC2 mutants posits these genes as potential candidates in the formation of autonomous endosperm development within *Hieracium*. The FIE protein is a core component of the PRC2 complex central to the regulation of *Arabidopsis* endosperm development and is also shown to affect *Hieracium* endosperm development (Rodrigues et al. 2008). In sexual *Hieracium *species downregulation of the *FIE *orthologue (*HFIE*) using RNAi does not induce autonomous endosperm initiation and results in failed endosperm cellularization and embryo arrest post-fertilization (Rodrigues et al. 2008). Arrest of embryo and endosperm formation is also observed in fertilized *Arabidopsis fie *mutants (Ohad et al. 1999). In apomictic *Hieracium *species *HFIE *downregulation results in an inhibition of autonomous embryo and endosperm formation, developing seeds arresting early with the endosperm failing to cellularize. However, cross-pollination in *HFIE* downregulated apomictic *Hieracium *lines results in restoration of endosperm cellularization that contrasts to what is seen in the fertilized sexual. In sexual species, defects in nuclear migration were also observed, with the SN of sexual gametophytes often failing to position adjacent to the egg cell nucleus, and syncytial endosperm nuclei showing abnormal spacing during early development (Rodrigues et al. 2008). These observations suggest that in *Hieracium,* HFIE activity may impact upon basal process such as trafficking or skeletal function in the CC, potentially via effects upon auxin production similar to the nuclear migration defects in *Arabidopsis* reported by Panoli et al. (2015).

HFIE proteins from both sexual and apomictic *Hieracium* species (termed HFIEsex and HFIEapo, respectively) show a high level of sequence conservation that suggests a similar capacity for interaction may exist between them, but they do show structural differences in comparison to the *Arabidopsis* FIE protein (AtFIE; Rodrigues et al. 2008). While AtFIE interacts with *Hieracium* RBR and MSI1 orthologues both the apomictic and sexually derived HFIE proteins fail to interact, suggesting interaction with other as yet unidentified proteins for functionality. Expression of HFIEapo in the CC of the *Arabidopsisfie* mutant is able to restore mitotic repression (Rodrigues et al. 2008). Both HFIE proteins are shown to interact with the *Arabidopsis* SET-domain protein, CURLY LEAF, and a curled-leaf phenotype similar to that observed in *Arabidopsis* is observed in *Hieracium* where HFIE is downregulated (Rodrigues et al. 2008). It is possible that residual HFIE expression in the *Hieracium* RNAi lines may still be sufficient to repress CC nuclear proliferation in the sexual species. However, these observations suggest that although the HFIEapo protein exhibits genuine polycomb group-like activity in the *Arabidopsis* CC, it may be recruited into different functional complexes in *Hieracium*, potentially operating in conjunction with additional regulators of endosperm development. In apomicts, deregulation of these additional interactors may lead to autonomous endosperm formation. Elucidation of the HFIE protein interactions taking place in sexual and apomictic *Hieracium* and the function of any associated complexes will be a valuable next step in investigating the regulatory mechanisms underlying fertilization-independent endosperm development.

In crosses between sexual and apomictic *Hieracium* lines, two plants showing autonomous endosperm development without parthenogenesis were recently identified (Ogawa et al. 2013). FGs in these plants are produced via the sexual pathway and require fertilization for embryo development but in the absence of fertilization, in both lines, ~18 % of ovules develop endosperm autonomously. Backcrosses of one of these autonomous endosperm lines to a sexual species show that the autonomous endosperm phenotype is inherited in a genetically dominant manner in progeny. Efforts are underway to map the *AutE* locus, and it will be interesting to learn whether this locus is tightly linked to *LOP*. *AutE* germplasm provides a valuable resource for isolating and investigating signalling events involved in fertilization-dependent and fertilization-independent endosperm initiation and early seed development. Observations of auxin-induced endosperm initiation in *Arabidopsis* raise the possibility that similar signalling pathways are active in *Hieracium* (Figueiredo et al. 2015). If auxin signalling does play a part in triggering *Hieracium* autonomous endosperm development, its origins are likely to be different to the paternal gene expression responsible for the signal in *Arabidopsis,* due to the lack of any paternal genetic contribution in apomictic species. Auxin appears to be a single component of multiple positive and negative signals that are together required for successful endosperm initiation and subsequent development in *Arabidopsis*.

Once initiated, cellularization is the hallmark of functional endosperm formation. Autonomous endosperm initiated in *Arabidopsis* FIS-class mutants or in response to elevated CC auxin levels does not mature and fails to cellularize, showing that additional signalling inputs required for complete development are absent. The autonomous endosperm produced in *Hieracium* AutE lines develops to completion in the absence of paternal genetic information or embryo-derived signals, both of which are required for correct endosperm development in *Arabidopsis* (Aw et al. 2010; Lafon-Placette and Kohler 2014). Ovules successfully mature and set seedless fruit indicating that endosperm-derived signals alone are sufficient to drive this process in *Hieracium*. The physical and genetic separation of autonomous endosperm development in *AutE* germplasm offers unique opportunities for dissection and functional characterization of the regulatory pathways associated with endosperm development. As new information emerges on the role of phytohormones, peptides and other molecular signalling pathways involved in endosperm initiation the use of new reporters and sophisticated transcriptomic analyses to exploit this *Hieracium* resource will provide a valuable comparative approach. Physiological analyses of *Hieracium* autonomous endosperm will provide insight to key features and processes essential to endosperm development while transcriptomic comparisons of fertilization-dependent and autonomously developing endosperm will provide valuable insight to the molecular pathways essential to endosperm initiation and development and opportunities for identification of causal genes at the AutE locus.

## Summary and outlook

The developmental patterning and events taking place during late FG development and early endosperm development are highly conserved across the angiosperms, and it is natural to suppose that the underlying mechanisms are equally conserved. Recent research provides some indication of diversity amongst the molecular mechanisms and the functioning of their components to achieve the same goals in endosperm development. It is clear that PcG proteins are well conserved amongst plants, with all multicellular plants having a functional PcG system that plays a number of important roles in regulating developmental transition (Hennig and Derkacheva 2009; Mozgova et al. 2015). In *Arabidopsis,* the FIS-PRC2 plays a central, well-defined role in multiple stages and processes including CC mitotic repression, endosperm cellularization, and polytubey blockade. Poly-functionality appears to be an intrinsic capacity of the PRC2, and a complex model for its function and composition is emerging in various model and crop plant species (Tonosaki and Kinoshita 2015; Hennig and Derkacheva 2009). As we gain a wider view of endosperm development and the extent of differences in regulatory pathways governing initiation between species, the importance of organism specific investigations is clear where mechanisms appear to have evolved independently.

Recent research reveals new information on the underlying genetics, signals, and checkpoints regulating endosperm initiation. The identification of auxin as a dominant signal initiating endosperm development in *Arabidopsis* raises questions about the conservation of a positive signal responsible for endosperm initiation in other species and its implications in efforts to engineer apomixis. Cell and nuclear fusion have significant roles in plant reproductive development that require further investigation and understanding. Differences in nuclear behaviour between monocots and dicots prior to fertilization suggest additional modes of pre-fertilization mitotic suppression may exist between them. For example, the partial fusion of the PN in monocots prior to fertilization may provide an additional or alternative safeguard against nuclear proliferation until the arrival of the sperm cell which provides the necessary cues for complete fusion and subsequent initiation of nuclear mitosis, potentially also explaining the limited examples of complete fertilization-independent endosperm development observed amongst monocots as compared to dicots. Although endosperm initiation with arrival of the paternal genome is common to all sexually reproducing species, the nature of the paternal factors involved for different species is an important question. Knowledge of cereal endosperm development will deliver the greatest practical impacts and is the obvious ultimate target of future research. Comparative investigations using monocot systems along with translating knowledge of autonomous development from apomictic *Hieracium* will be keys to ongoing research and successful genetic engineering approaches for resilient and fertilization-independent seed crops.
